# Intense pulsed-light treatment improves objective optical quality in patients with meibomian gland dysfunction

**DOI:** 10.1186/s12886-023-02939-9

**Published:** 2023-04-28

**Authors:** Woong-Joo Whang, Jeongseop Yun, Kyungmin Koh

**Affiliations:** 1 Department of Ophthalmology, Yeouido St. Mary’s Hospital, The Catholic University College of Medicine, Seoul, Republic of Korea; 2 Department of Ophthalmology, Kim’s Eye Hospital, Konyang University College of Medicine, Seoul, Republic of Korea

**Keywords:** Dry eye disease, Intense pulsed light, Meibomian gland dysfunction, Optical quality analysis, Quality of vision

## Abstract

**Background:**

To evaluate changes in objective optical quality following intense pulsed light (IPL) treatment combined with meibomian gland (MG) expression (MGX) in patients with MG dysfunction (MGD).

**Methods:**

This retrospective cross-sectional study included MGD-related dry eye disease (DED) patients who received IPL treatment between March and December 2021 at Kim’s Eye Hospital, Seoul, Republic of Korea. Each patient underwent four sessions of IPL treatment using Lumenis M22 (Lumenis Ltd., Yokneam, Israel) and MGX at three-week intervals.

**Results:**

This study included 90 eyes from 45 patients with MGD. The mean age was 52.3 ± 16.1 years (range, 20–75 years), and 53.3% (24/45) of patients were female. Compared with the baseline, all clinical symptoms and signs significantly improved after IPL treatment combined with MGX. All optical quality parameters obtained with an optical quality analysis system (OQAS: Visiometrics, Castelldefels, Spain) have improved significantly over the baseline (p < 0.001).

**Conclusions:**

In patients with MGD, IPL treatment combined with MGX improved the objective optical quality and clinical signs and symptoms of DED.

**Supplementary Information:**

The online version contains supplementary material available at 10.1186/s12886-023-02939-9.

## Introduction

Dry eye disease (DED) caused by meibomian gland (MG) dysfunction (MGD) is a common disorder [1]. The prevalence of MGD in the general population is estimated to be 30.5 to 68.3% [2]. It is a multifactorial disorder that causes symptoms such as visual disturbance and ocular discomfort, in addition to instability of the tear film [3]. Tear film plays a vital role in ensuring good visual quality and preserving the decent optical quality [4, 5].

Well-maintained meibum lipids in tear film prevent evaporation and provide a smooth optical surface [6]. In patients with MGD, the meibum is more viscous and cannot properly seal the tear film on the eye to prevent evaporation [7]. Blurry vision associated with DED may be linked to increased optical aberrations that reduce the optical quality [8]. Another study assess the effect of eyedrops found they improved optical quality in DED patients [9]. Changes in tear film in the DED can cause irregularities in the corneal surface, and the DED exhibits an irregular distribution of tear film in the cornea [10]. This means that DED have more optical aberration than normal eyes [11].

In recent years, intense pulsed light (IPL) treatment has emerged as a useful treatment option for DED with MGD [12–15]. Numerous studies have documented significant improvements in parameters such as the ocular surface disease index (OSDI) score, corneal and conjunctival staining (CFS) score, Schirmer test value, meibomian gland expressibility (MGE), and tear break-up time (TBUT) following IPL treatment [15–19]. Additionally, improved subjective visual acuity (VA) and the quality of vision after IPL treatment has been reported in patients with DED [20, 21]. However, improvement in the objective optical quality following IPL treatment has not yet been evaluated. In this study, we investigated the effects of IPL treatment on the objective optical quality and symptoms of DED in patients with MGD.

## Methods

### Subjects

This study was approved by the Institutional Review Board of Kim’s Eye Hospital, Seoul, Republic of Korea (2021-09-006) and adhered to the tenets of the Declaration of Helsinki. We conducted a retrospective cross-sectional study on MGD-related DED patients who received IPL treatment between July and December 2021 at Kim’s Eye Hospital, Seoul, Republic of Korea. Considering the retrospective nature of the study and the use of de-identified patient data, the requirement for written informed consent was waived by the Institutional Review Board of Kim’s Eye Hospital, Seoul, Republic of Korea. The inclusion criteria were adults aged 20–80 years who met all three of the following criteria: OSDI score ≥ 13 points [22]; TBUT < 10 s for both eyes [23, 24]; having at least two clinical signs linked to MGD: redness or thickening of the lid margin, telangiectasia, reduced or nonexistent secretions, poor quality secretions, and MG capping [25, 26]. The exclusion criteria were patients with contraindications to IPL (recent tanning, skin diseases, active ocular infection, inflammation, and allergies); patients currently using eye drops other than artificial tears; those with evident scarring or severe keratinization of the eyelid margin; and patients with a history of ocular surgery or trauma, punctal plug insertion, heat treatment, MGE, and autoimmune diseases such as Sjögren’s syndrome.

### Examinations

The primary endpoint was the change in the objective optical quality, which was analyzed using an optical quality analysis system (OQAS: Visiometrics, Castelldefels, Spain). The secondary endpoints were as follows.

Changes in the best-corrected distance VA (BCVA), OSDI score, lipid layer thickness (LLT), partial blinking rate (PBR), TBUT, CFS score, Schirmer I test value, and MGE. To avoid affecting test results, the tests were conducted in the less invasive to more invasive order. Consequently, BCVA, OSDI, OQAS, LLT, PBR, TBUT, CFS, Schirmer I test, and MGE were tested in this order. All of the above tests were conducted by two ophthalmologists (JK and KK) prior to treatment and three weeks after four IPL treatment sessions were completed.

BCVA was assessed according to a standardized protocol following manifest refraction assessment in both eyes. The Early Treatment of Diabetic Retinopathy Study chart was placed 4 m from the patient in a standard light box [27]. BCVA results were converted into the logarithm of the minimum angle of resolution (logMAR) for statistical analysis.

To estimate the magnitude of patients’ discomfort, participants were requested to complete the OSDI questionnaire containing 12 questions before and after receiving IPL treatment [28, 29]. OSDI score ≥ 13 is used to define symptomatic DED [30, 31]. Therefore, only patients with an OSDI score ≥ 13 were included in the study.

**Fig. 1:**
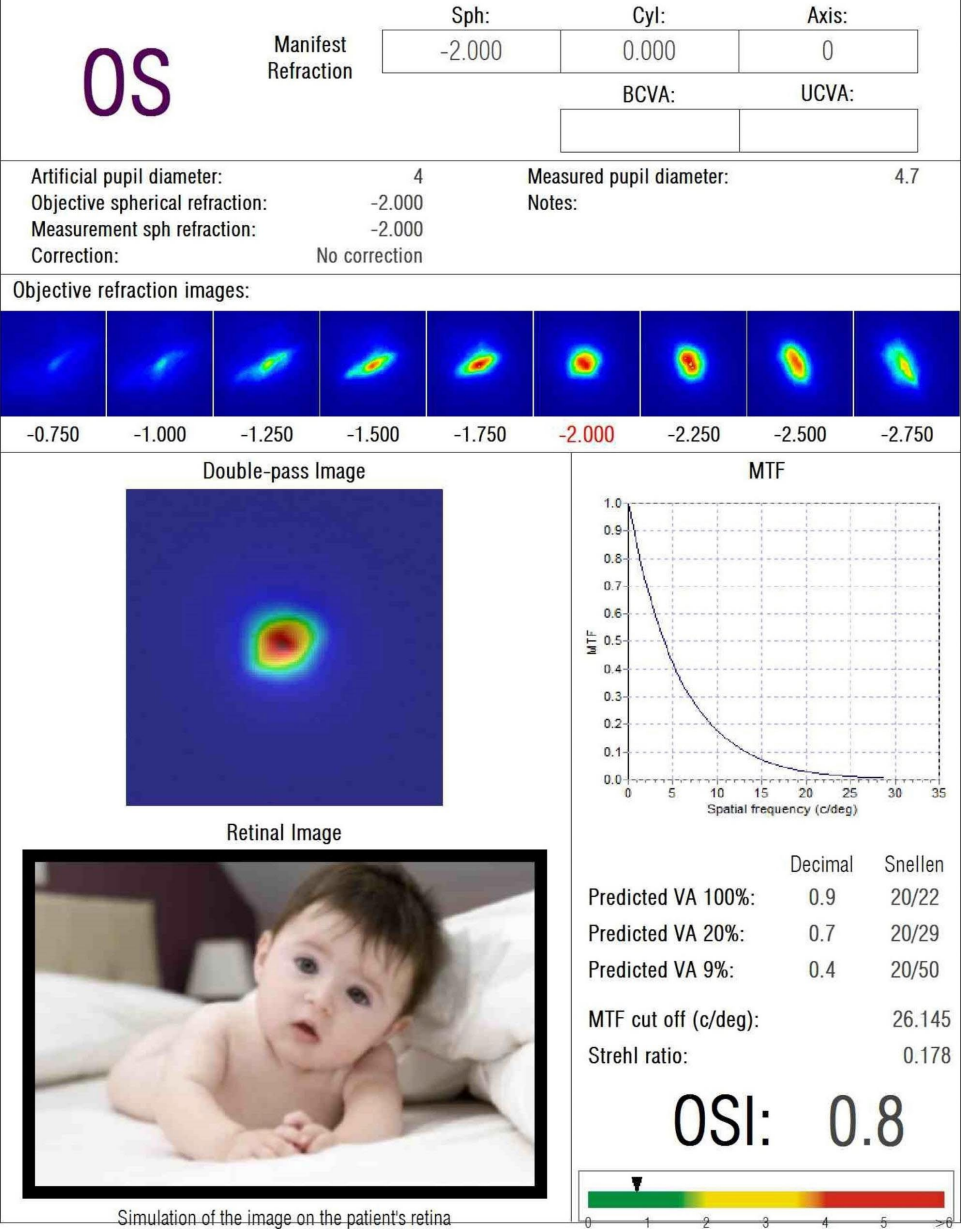
Image about the post-treatment objective optical quality parameters evaluated by optical quality analysis system (OQAS®, Visiometrics, Castelldefels, Spain) of a 61-years old female patient. It indicates objective scatter index (OSI) 0.8, MTF cut-off 26.145, and Strehl ratio 0.178

OQAS measurements were performed by an ophthalmologist (JK) on both eyes of each patient under low light conditions with a pupil diameter of 4.0 mm, prior to instillation of eye drops, as recommended by the manufacturer [32]. OQAS uses a double-pass (DP) technique to objectively measure the image formed on the retina by fusing quantified optical aberrations owing to the diffusion of light in both directions caused by the loss of eye transparency [32, 33]. OQAS measures three parameters: objective scatter index (OSI), modulation transfer function (MTF) cutoff, and point spread function (PSF) expressed as the Strehl ratio (SR) (Fig. 1). OSI objectively quantifies scattered intraocular light [34]. The OSI is defined as the ratio of the light embedded in the peripheral ring to the center crest of the DP image. It depicts the impact of aberration and diffusion on the DP image [35].

The MTF indicates a quantified visual acuity value, and the MTF limit is the spatial rate at which the MTF declines to zero. MTF is a widely agreed upon and validated parameter for quantifying image quality of intraocular lenses (IOLs) [36–38]. The higher the value, the clearer is the optical quality. The MTF cut-off is the cut-off point on the x-axis of the MTF curve, which can be directly calculated from the PSF [39]. The PSF describes the quality response of an imaging system and is indicated by the SR, with a value of 1 suggesting a perfect optical system [40]. The higher the value, the clearer the optical quality [41].

LLT and PBR measurements were performed using the LipiView® II ocular surface interferometer (LVII: TearScience Inc., Morrisville, NC, USA [42]. LVII automatically detects and analyzes blink rate and quality [43].

It shows the number of complete and incomplete blinks and numeric blinking frequency. The partial blinking is defined as blinking without contact of the upper and lower eyelids [44].

To measure TBUT, a drop of non-preserved saline solution was added to a fluorescein strip (Haag-Streit Köniz, Switzerland) that was applied to the lower palpebral conjunctiva. Participants were asked to blink several times for few seconds to ensure adequate mixing of the dye on the cornea. Then, the eye was checked using a slit lamp (Haag-Streit BP 900; Haag-Streit, Köniz, Switzerland) with maximum cobalt blue light. The participants were asked to open their eyes wide and look straight, and a single ophthalmologist (KK) measured the time taken for a black spot or line to appear on the cornea. Interval between last blink to the first sign of tear film rupture was recorded as TBUT [45, 46]. While measuring TBUT, the CFS score (0–9) was also evaluated [47].

The Schirmer I test was conducted without anesthesia using sterile Schirmer strips. Strips were placed in the center of the lower fornix for 5 min [48]. During this process, patients were asked to close their eyes. The length of the wet section of the tape was recorded in millimeters, and the test was considered positive if wetting of the paper was ≤ 5 mm [49].

MGE was measured from the five central glands of the upper and lower eyelids with compression forceps (Katena Products, Parsippany, NJ, USA) [28]. Only one experienced ophthalmologist (KK) applied compression.

The induced meibomian secretion (meibum) assessed from 0 to 3 points as follows: 0, clear meibum; 1, cloudy meibum on mild compression; 2, cloudy meibum on moderate compression; and 3, no meibum or toothpaste-like meibum expressed through intense compression [24, 50]. A higher MGE score is indicative of a more obstructive meibum secretion [51, 52].

### IPL treatment protocol

A single ophthalmologist (JY) managed Lumenis M22 (M22: Lumenis Ltd., Yokneam, Israel) throughout the study. All patients received four IPL treatment sessions at 3-week intervals. Both eyes were treated on the same day, with the right eye being treated before the left eye. The therapeutic process was based on a previously published technique [53]. Topical 0.5% proparacaine anesthetic eye drops (Paracaine; Hanmi Pharm, Seoul, Republic of Korea) were instilled in each eye. After applying a thin (approximately 1 mm) coat of pre-cooled ultrasound gel to the skin of the eyelids including the nose, the Jaeger lead plate (Katena Products, Denville, NJ, USA) was placed in the conjunctival sac to protect the eye. A series of 20 overlapping pulses were applied to the skin in the preauricular area and across the cheeks and nose on each side. This process was repeated twice. IPL treatment was performed using a 590-nm filter with a 6 mm cylindrical light guide [54]. The fluence was determined based on Fitzpatrick skin types (13–19 J/cm^2^), as described in earlier studies [54–56]. MGX was performed on the upper and lower eyelids using eyelid compression forceps (Katena Products, Parsippany, NJ, USA) immediately following IPL treatment. All patients were administered a preservative-free carboxymethylcellulose sodium 0.5% solution (Refresh Plus®; Allergan Inc., Irvine, CA, USA) six times a day.

### Statistical analyses

Data were analyzed using the IBM Statistical Package for the Social Sciences (SPSS) version 22 (Chicago, IL, USA), and statistical significance was set at p < 0.05. Continuous variables are presented as the mean ± standard deviation. Paired analysis was used to compare the pre- and post-treatment data. Normality of the data was assessed using the Kolmogorov–Smirnov test. All paired analyses showed nonparametric distributions and were performed using the Wilcoxon signed-rank test.

## Results

This retrospective study included 90 eyes from 45 patients with MGD. Each eye underwent four IPL treatment and MGX sessions at 3-week intervals. The mean age was 52.3 ± 16.1 years (range, 20–75 years), and 53.3% (24/45) of the patients were female. The objective optical quality and dry eye parameters improved significantly after the IPL treatment. Table 1 summarizes the clinical signs and symptoms before and after IPL treatment combined with MGX. No systemic or skin-related side effects or ocular complications were noted.

**Table 1:** Changes in clinical parameters for patients with meibomian gland dysfunction between before and three weeks after four sessions of intense pulsed light treatment combined with the meibomian gland expression

Parameter	Pre-Treatment	Post-Treatment	*P*-value
BCVA (logMAR)	0.04 ± 0.07	0.03 ± 0.05	0.08
Optical quality parameters			
OSI	3.37 ± 3.05	1.45 ± 0.92	< 0.001
MTF cutoff	21.06 ± 12.30	31.62 ± 9.58	< 0.001
Strehl ratio	0.12 ± 0.06	0.18 ± 0.04	< 0.001
Lipid layer thickness (nm)			
Mean	75.30 ± 25.03	82.73 ± 20.51	0.031
Maximum	89.48 ± 18.70	95.00 ± 12.37	0.021
Minimum	61.90 ± 28.73	67.64 ± 25.92	0.207
Partial blinking rate	0.51 ± 0.42	0.39 ± 0.40	0.037
MG expressibility	2.06 ± 0.72	0.92 ± 0.64	< 0.001
TBUT (sec)	3.18 ± 1.46	6.60 ± 1.96	< 0.001
CFS score	1.18 ± 0.82	0.61 ± 0.55	< 0.001
Schirmer I test (mm)	8.41 ± 1.69	9.64 ± 1.72	< 0.001
OSDI score	31.46 ± 6.29	23.26 ± 5.96	< 0.001

The results are expressed as mean and standard deviation. BCVA, best corrected visual acuity; OSI, objective scatter index; MTF, modulation transfer function; logMAR, logarithm of the minimum angle of resolution; MG, meibomian gland; Corneal and conjunctival fluorescein staining (scale from 0 to 9); TBUT, tear break-up time; CFS, OSDI, ocular surface disease index questionnaire

All optical quality parameters improved significantly after treatment (all p < 0.001). The baseline OSI, MTF cutoff, and SR improved from 3.37 ± 3.05, 21.06 ± 12.30, and 0.12 ± 0.06, to 1.45 ± 0.92, 31. 62 ± 9.58, and 0.18 ± 0.04, respectively. Following treatment, BCVA showed a significant improvement from logMAR 0.04 ± 0.07 to logMAR 0.03 ± 0.05 (p = 0.08). Mean and maximum LLT showed significant improvement after treatment. The baseline mean, maximum, and minimum LLT improved from 75.30 ± 25.03, 89.48 ± 18.70, and 61.90 ± 28.73 to 82.73 ± 20.51, 95.00 ± 12.37, and 67.64 ± 25.92. A significant decline in PBR from 0.51 ± 0.42 to 0.39 ± 0.40 was observed after treatment (p = 0.037). The baseline MGE, CFS, Schirmer I test (mm), TBUT (s), and OSDI score improved from 2.06 ± 0.72, 1.18 ± 0.82, 8.41 ± 1.69, 3.18 ± 1.46, and 31.46 ± 6.29 to 0.92 ± 0.64, 0.61 ± 0.55, 9.64 ± 1.72, 6.60 ± 1.96, and 23.26 ± 5.96, respectively. All these parameters improved significantly after treatment (all < 0.001).

## Discussion

MGD is a common ocular surface disease and is one of the most common diseases encountered in ophthalmology [18]. Obstructive MGD is the most common reason for low meibum delivery [49]. The obstruction is accompanied by thickening and opacification of the expressed meibum, which blocks the orifices [57]. Irregularities in the air–film interface on the ocular surface contribute to fluctuating vision owing to the scattering of ocular light measured by the double-pass imaging system, thereby degrading image quality [58, 59]. MGD can cause ocular discomfort, decreased VA, and decreased quality of vision [60]. IPL can heal the MG, enabling it to produce good quality meibum [61]. It is expected that IPL treatment will play a positive role in maintaining the tear film and improving VA and clinical outcomes in patients with MGD. The Tear Film & Ocular Surface Society Dry Eye Workshop II Management and Treatment Report proposed IPL as second-stage treatment following education, eyelid hygiene, and various types of eye lubricants [62]. IPL treatment has proven to be highly promising for DED with MGD [63]. This is probably because the heat distribution alleviates abnormal secretions of glands, kills harmful Demodex mites, and reduces inflammatory markers on the ocular surface [64]. Regarding the mechanism of action of IPL in MGD, the treatment heats the MGs by increasing the temperature of the thin periocular skin, favoring the melting of the meibum [65]. Tear lipids secreted by decent MG helps maintain the solidity of the tear film [66].

Visual complaints due to DED is often clinically overlooked due to its sometimes diurnal variation [67], fluctuating visual morbidity nature as traditionally measured [68]. The irregularities in the tear film increase ocular scattering, exacerbating contrast sensitivity and optical quality [57, 69]. Pathological thinning of the tear film has been documented to significantly affect the light coming into the pupils and influence the subjective quality of vision (QOV) [70]. An unstable tear film may weaken visual function, resulting in blurred vision and glare [71].

The irregularity of the tear film may cause an uneven corneal surface and, consequently, subject the retina to larger optical aberrations [11]. Tear film instability also reduces both contrast sensitivity and VA [4, 72]. The OSI is not only related to the intraocular scattering caused by cataract, but also to the stability of the tear film [73]. Several studies have found that the OSI is higher in eyes with DED than in normal eyes [57, 69, 74]. The IPL treatment appears to have improved the stability and evenness of the tear film, leading to an improvement in BCVA and objective optical quality in our study. According to another study, similar to ours, IPL treatment can significantly improve tear stability, subjective OSDI, and subjective QOV scores [20]. Specifically, the results of this study indicate that visual disturbances related to glare, halos and blurred vision have markedly improved.[20].

The strength of our study is that it is the first study to show the improvement in objective optical quality after combined IPL treatment and MGX for MGD. In this study, BCVA and objective optical quality improved significantly from baseline after four sessions of IPL treatment combined with MGX.

There are limitations to the interpretation of our findings. First, this study was limited by its uncontrolled design and small number of participants. Second, because this study used a before- and after-treatment observational design, no controls were enrolled. This may have introduced bias into the efficacy findings. It would make the study more credible if the test results were included after each IPL treatment rather than only the baseline and final treatment. As the IPL treatment sessions are relatively long, 3 weeks apart, changes in the indicators after each IPL treatment would have been very informative. Third, in addition to IPL treatment, patients in this study used artificial tears. It could not be inferred from this study how the ocular surface parameters and optical quality would change with artificial tears alone. Fourth, There was no assessment of eyelid margins or MG dropout, which would provide additional information on MG changes. Fifth, the last follow-up was completed three weeks after the completion of all treatment sessions. Thus, the observation period was insufficient to determine the long-term effects of IPL treatment combined with MGX. Because the subjective results have been reported in many other articles, our main purpose was to focus on changing the objective optical quality. To validate the current findings, a prospective case–control study comparing patients with and without IPL treatment (artificial tears only) with a larger number of participants and longer follow-up is necessary.

In summary, IPL treatment combined with MGX improved the objective optical quality and DED parameters in patients with MGD. Based on our findings, we hope that the indications and extent of IPL use can be expanded.

## Electronic supplementary material

Below is the link to the electronic supplementary material.

Supplementary Material 1

## Data Availability

The datasets used and/or analyzed during the current study available from the corresponding author on reasonable request.
